# Maltreated Children Use More Grammatical Negations

**DOI:** 10.1007/s10826-017-0905-3

**Published:** 2017-10-25

**Authors:** Franziska Knolle, Claire D. Vallotton, Catherine C. Ayoub

**Affiliations:** 1 Department of Psychiatry, University of Cambridge, Cambridge, UK; 2 Human Development & Family Studies, Michigan State University, East Lansing, MI USA; 3 Harvard Medical School & Massachusetts General Hospital, Boston, MA USA

**Keywords:** Childhood maltreatment, Abuse, Language acquisition, Negativity bias, Resilience

## Abstract

Many studies reveal a strong impact of childhood maltreatment on language development, mainly resulting in shorter utterances, less rich vocabulary, or a delay in grammatical complexity. However, different theories suggest the possibility for resilience—a positive adaptation to an otherwise adverse environment—in children who experienced childhood maltreatment. Here, we investigated different measures for language development in spontaneous speech, examining whether childhood maltreatment leads to a language deficit only or whether it can also result in differences in language use due to a possible adaptation to a toxic environment. We compared spontaneous speech during therapeutic peer-play sessions of 32 maltreated and 32 non-maltreated children from the same preschool and equivalent in gender, age (2 to 5 years), home neighborhood, ethnicity, and family income. Maltreatment status was reported by formal child protection reports, and corroborated by independent social service reports. We investigated general language sophistication (i.e., vocabulary, talkativeness, mean length of utterance), as well as grammatical development (i.e., use of plurals, tense, grammatical negations). We found that maltreated and non-maltreated children showed similar sophistication across all linguistic measures, except for the use of grammatical negations. Maltreated children used twice as many grammatical negations as non-maltreated children. The use of this highly complex grammatical structure shows an advanced linguistic skill, which shows that childhood maltreatment does not necessarily lead to a language deficit. The result might indicate the development of a negativity bias in the structure of spontaneous language due to an adaptation to their experiences.

## Introduction

The effects of maltreatment on the child’s development are widespread, particularly when a caregiver is the perpetrator, compromising the caregiver-child relationship (Cicchetti and Toth 2005; Garmezy 1983; Teicher et al. 2016). Affected developmental outcomes include behavioral (Ethier et al. 2004; Valentino et al. 2011), emotional (Sullivan et al. 2008; van Harmelen et al. 2010), cognitive (Ayoub et al. 2006; Harpur et al. 2015), and language domains (Coster and Cicchetti 1993; Sylvestre et al. 2016).

Many studies have shown that language development is facilitated when caregivers’ language input is responsive, engaging, and contingent to the child’s cues (Baldwin et al. 1996; Zimmerman et al. 2009). In families in which children experience maltreatment, however, language input may be adversely affected, which may in turn negatively impact the child’s language development (Sylvestre et al. 2016). Examining the differences in the language used by abusive or neglectful parents during parent-child interaction, a recent meta-analysis (Wilson et al. 2008) showed that maltreating parents show less active, responsive and positive, but more aversive language interaction with their children. Eigsti and Cicchetti (2004) investigated the effect of the mother’s language input on the child’s language performance. They found qualitative differences in the maltreating parent’s language input (i.e. fewer utterances all together; fewer questions; fewer complex sentence structures) to their children (average age 5 years) and a delay in the children’s overall vocabulary and production of syntactic structures.

Similarly, Coster et al. (1989) investigated the impact of maltreatment on aspects of language development in a group of 2-year-olds. They found delays in syntactic development; in particular, on average maltreated children had a shorter mean length of utterance (MLU), that is, the average word length of a spoken sentence or sentence fragment. They also identified deficits in expressive language, meaning that the language the children actively used showed a reduced lexical diversity (i.e., vocabulary diversity). However, maltreated children did not show deficits in receptive language, meaning that the children’s abilities to understand the content and respond appropriately was maintained. Relatedly, Beeghly and Cicchetti (1994) found that maltreated 2.5-year olds used fewer words and fewer word types to refer to their internal state than a matched control group; both groups were drawn from the same urban, low-income area and matched in age and gender. Furthermore, Prasad et al. (2005) found in a group of physically abused children (1–6-year-olds) delays in receptive and expressive language. Although, a few studies indicate that childhood maltreatment might not necessarily lead to a language deficit (Flisher et al. 1997; MacFayden and Kitson 1996), it seems to be widely agreed that maltreatment has a strong impact on speech production when assessed in standardized language tasks.

Along with the potentially different effects of the different maltreatment types, moderated by the child’s stage of development and other specific environmental conditions, children themselves have their unique ways of coping with and adapting to their environments. Thus, there is no singular child maltreatment syndrome (Horton and Cruise 2001), but rather a range of effects influencing various domains of children’s development. In contrast to the idea that maltreatment may only cause a deficit in language and possibly general cognition (Scarborough et al. 2009; Veltman and Browne 2001), different studies show that children are resilient in the face of adversity. The Buffering Hypothesis (Cohen and Wills 1985) states that the availability and use of social support through friends but also social workers or therapeutic staff after experiencing any form and severity of adversity can moderate the long lasting negative effects of stress, including childhood maltreatment. In relation to childhood maltreatment studies showed that social support is a significant moderator of long-term consequences, such as anxiety or depression of childhood maltreatment (Evans et al. 2013; Sperry and Widom 2013).

Similarly, the Dynamic Skills Theory (Fischer and Bidell 2006) posits that children can build strategies to adapt to their individual environments, including negative ones, and develop along alternate but equally sophisticated pathways. It furthermore proposes that maltreated children develop biases in their cognition and emotion reflective of their negative experiences and indicative of a negative world-view; this bias is directly expressed through their behavior and language (Ayoub et al. 2006; Fischer and Bidell 2006). Ayoub et al. (2006) have elaborated the biasing idea using a language approach by analyzing children’s abilities to use language to represent increasingly sophisticated social concepts by re-telling a series of positively and negatively themed stories. They used children from two and a half- to 5-years old. Results showed that although maltreated children had lower levels of complexity than non-maltreated children for the positive stories, they had the same or greater complexity in their stories compared to non-maltreated children when retelling negative stories. Interestingly, the two groups reached similar levels of overall complexity as maltreated children often transformed positive stories into negative versions (Ayoub et al. 2006). This behavior indicates that maltreated children develop a negative bias, which is shown through language using negative concepts of behaviors, emotions, and cognitive processes, and which we expect may be directly expressed in the content of their language. However, this study did not investigate the grammatical structure of the language, therefore we do not know whether these children used highly complex grammatical negations to express this negativity bias. The answer to this question would nicely link to the idea that language and language use shapes our thinking, impacts our world view and self-perception (Chen et al. 2014; Cibelli et al. 2016).

Grammatical negations, for example ‘I don’t cry when I kick people.’ (Statement of a 4-year-old boy of this sample) represent a sophisticated language skill (Klima and Bellugi 1966; Déprez and Pierce 1993; Thornton and Rombough 2015), which usually develops between age 2 and 3 (Cameron-Faulkner et al. 2007). It requires the child to use the negation particle in a fixed syntactic position which leads to restructuring of the sentence, applying the appropriate auxiliary (e.g. will, might, can, do, be, would, etc.), and using the correct contracted form (e.g. do + not = don’t; will + not = won’t; would + not = wouldn’t). The use of grammatical negations in spontaneous speech does not only show an advanced language sophistication, but it possibly also shows a potential negative bias that develops due to the child’s adaptation to her/his toxic environment (Linebarger 1987; Szabolcsi 2004) as described above.

The overarching question of our study was whether childhood maltreatment would always lead to a language delay, or whether it could also lead to a difference in language use. In order to answer this question, we examined the impact of corroborated (i.e., documented and verified as described below) childhood maltreatment on language sophistication of spontaneous speech by exploring possible deficits or differences in a set of general language sophistication and specific grammatical measures. We hypothesized (I) that maltreated children would exhibit reduced overall language skill, but (II) that they would be showing an enhanced use of grammatical negations which would on the one hand indicate an advanced skill and on the other hand indicate a negativity bias.

## Method

### Participants

The participants were 64 children between 2 years and 5.5 years old (mean: 46 months; SD: 10.6 months) who all attended the same preschool program. Half of the children (12 female, 20 male; mean age: 46.5 months, SD: 12.1 months; races: white: 23, Afro-American: 3, Hispano-American: 2, bi-racial: 2, other: 1, missing data: (1) had experienced childhood maltreatment as assessed based on formal child protection reports, which were additionally confirmed by the preschool center’s social service reports, and the other half of the children had been identified as non-maltreated (17 female, 15 male; mean age: 47 months, SD: 9.5 months; races: white: 22, Afro-American: 5, Hispano-American: 0, bi-racial: 3, other: 2, missing data: 0).

The sample selected for the current study was drawn from two sets of raw video data originally collected for different studies using overlapping groups of children within the same preschool center. Both studies used an identical therapeutic play setting, with peer dyads of similar age chosen from the same preschool center in an urban, low-income neighborhood in the Northeastern United States. Furthermore, all children came from low socio-economic households (<$16500 per annum for a min. of three people), and the non-maltreated group was selected from children in the same preschool to approximate the maltreated group regarding child age, gender, and racial-ethnic group membership. For more details on the two studies see the Developmental Pathways Project (Ayoub et al. 2006) and the stress reactivity study (Rappolt-Schlichtmann 2007). The two studies a total of 210 participants; 68.3% male, 31.7% female, age from 22 to 73 months. The sample used for this study was selected based on the age (≥2 year), dyad structure (i.e., similar age, ±2 months), perpetrator (i.e., primary caregiver), time point of last reported incidence of maltreatment (i.e., not longer than 5 month ago), and months in therapy (≤2 months).

### Procedure

All children in the current study participated in therapeutic peer-play sessions, regardless of maltreatment status. All children were chosen for participation in therapeutic play sessions exhibited low social skills. For each observation, two children at a time were taken into a special play room with a variety of age-appropriate toys (e.g. play dough; kitchen set; doll house; cars). During these play sessions, the children were supervised and monitored by two adult therapists (or therapy interns). If necessary they initiated and stimulated the play between the children, to promote social interaction between the children and help them each build basic social skills, such as initiating and joining play, and communicating about disagreements instead of solving problems physically. The duration of each play session ranged from 30 to 60 min (see Ayoub et al. 1996, for additional information about pair play therapy) during which they were videotaped.

The age difference within one dyad never exceeded 2 months. Prior to the first therapeutic play session, the dyads of playmates were not familiar with each other, though they might have met briefly in the preschool center before. Since maltreatment has several corollaries which also influence children’s development, the assessment of the true impacts of maltreatment on development is very difficult. We, therefore, compared all measures of maltreated children to non-maltreated peers who experienced similar familial and neighborhood environments and the same demographic risks (Sattler 1998; Tzeng et al. 1991).

### Measures

#### Demographic information

The information was provided by the child’s guardian upon the child’s enrollment in the preschool program, and was cross-checked with any information found in children’s records kept by their social workers. Additionally, we registered data relevant for covariance analyses, such as the intake age of the child into the therapeutic program, and the dyad partner’s age, gender, and maltreatment status. The dyad partners were randomly assigned. Of the 32 dyads of playmates in this study, seven were mixed in gender, but had the same maltreatment status. Seven were mixed in the maltreatment status but were matched with regard to gender. One dyad of playmates was mixed in both gender and maltreatment status. The age difference within one dyad never exceeded 2 months.

#### Linguistic measures

*General language development*: To assess the children’s general language sophistication, we created a set of variables for general language measures, including mean length of utterance (MLU), type-token-ration (TTR), talkativeness (number of all word occurrences (tokens) per minute), and lexical diversity (number of different words (types) per minute). MLU describes the number of items in a single utterance. TTR which is the relationship between the total number of different words and the total number all word occurrences is measure of general lexical variation and active vocabulary.

*Grammatical language measures*: To assess the children’s grammatical skills, we observed uses of plurals, past and present tense, and auxiliaries, and grammatical negations (see Table 1 for detailed description examples, and descriptive statistics). In terms of grammatical negations, we counted cases of morphosyntactic negation, which requires an auxiliary verb and a negation particle. This type of negation leads to syntactic restructuring of the sentence, which is not the case when using morphosemantic negations such as ‘nobody’ or ‘nothing’ as those are equivalent to ‘everyone’ or ‘anything’. Therefore, the use of morphosyntactic negation rely on advanced grammatical abilities about syntactic positioning of the element within the sentence, use of auxiliaries, clipping of auxiliaries (i.e., do not - > don’t). We counted the use of ‘not’ or negated auxiliaries such as don’t, isn’t, aren’t, didn’t, won’t, haven’t, hasn’t, and doesn’t. ‘No’ was only included when it was used incorrectly instead of the grammatical negation particle ‘not.’ Since one-word negations (i.e., ‘no’) do not create sentence negation, we did not include them in the frequency of grammatical negations, but counted them in a separate variable as an expression of disagreement or denial. As negations can be used to convey a positive meaning (e.g., “I know I am not stupid!”), we assessed the meaning of all negations. We did not find any incident of a “positive negation”.

**Table 1:** Definitions, descriptions, and descriptive statistics for general language development, and grammatical sophistication

Variable names and definitions	Descriptions/examples	Descriptive statistics	
		Mean (SD) or %	Min-max
Talkativeness: Total number of tokens used per minute	For example, a child utters four sentences each five words long in 2 min; thus, the child utters 10 words per minute, i.e. (4 × 5)/2 = 10	16.07 (9.99)	0.12–45.88
Lexical diversity: Total number of word types used per minute	For Lexical Diversity, unique word types are counted and divided by the number of minutes; for example, ‘cat’ and ‘cats’ are considered the same word type, while ‘cat’ and ‘dog’ are considered two unique words	5.25 (2.51)	0.12–11.60
TTR: Type-Token Ratio is the total number of word types divided by total number of word tokens	Tokens describe the overall number of words spoken, including words that have been spoken more than once. Types count the number of unique words children use; for example, ‘want’, ‘wanted’, ‘cats’, and ‘cat’ are four tokens but only two types	0.38 (0.12)	0.19–1.00
MLU: Mean Length of Utterance is the total number of morphemes divided by total number of utterances	The utterance ‘Tony eat-s breakfast’ has a length of four morphemes, and ‘Jane is play-ing quiet-ly.’ has a length of six, thus the two utterances together have a mean length of five morphemes per utterance	3.34 (0.92)	1.00–5.99
MLU_R: As MLU, but with simple Yes/ No phrases removed from calculation		3.54(1.13)	1.00–7.28
Unique plurals: Number of unique plurals divided by number of unique word types	For Unique Plurals, each different type of plural is counted once; for example, ‘cats’ and ‘dogs’ are two unique plurals, but ‘cats’ and ‘cats’ is only one unique plural	0.02 (0.02)	0.00–0.07
Total plurals: Number of total plurals divided by number of total word tokens	Total Plurals counts all appearing plurals, for example, ‘cats’, ‘cats’, and ‘dogs’ are three total plurals	0.01 (0.01)	0.00–0.07
Third person-S: Total number of times the child uses third person-s divided by total number of tokens	Third Person-S describes the –s attached to the verb when using the third person; for example, ‘He plays tennis’ or ‘Does he sleep?’	0.01 (0.00)	0.00–0.03
Past tense: The total number of times the child uses past tense divided by total number of tokens	Past tense refers to a situation that lies back in time; for example, ‘Yesterday, I played football.’	0.07 (0.08)	0.00–0.55
Use of auxiliaries: Total number of auxiliaries divided by total number of word tokens	Auxiliaries can be used in questions (e.g. ‘Do you want some tea?’), tag-question (e.g. ‘He plays the piano very well, doesn’t he?’), in negations (e.g. ‘He didn’t like it.’) and in tense forms (e.g. ‘John is playing football.’)	0.02 (0.02)	0.00–0.07
Use of sentence negation: Total number of negative operator or determiners which created sentence negation, divided by the total number of utterances	Negative operator and determiners are, for example, ‘not’, ‘never’, ‘nobody’, and ‘nowhere’. This also includes the use of auxiliaries used in negations, for example, ‘don’t’, ‘won’t’, and ‘can’t’.	0.06 (0.05)	0.00–0.2
Use of no: Total number of times the child said ‘no’ divided by total number of word tokens	For example: ‘No hat!’, ‘No, I want this.’, ‘No, I don’t like the car!’	0.05 (0.06)	0.00–0.33

#### Maltreatment measures

Child maltreatment is a very complex phenomenon, with variation in types, severity, and frequency. Research literature generally distinguishes five types of child maltreatment which may all co-occur: emotional neglect, physical neglect, physical abuse, sexual abuse, and emotional maltreatment (Gilbert et al. 2009; Myers 1996). Furthermore, frequency and severity of maltreatment may exert differential influences on development.

Maltreated children in this study were identified through formal reports to the state-mandated child protection agency. The preschool center in which this study took place had a collaborative relationship with the children’s social workers, and maintained records on the children’s maltreatment status. The reports of maltreatment were identified as substantiated cases by the mandated agency and confirmed through the preschool centers’ social service reports. Perpetrators were primary caregivers. The maltreated children were then enrolled in therapeutic day care, 5 days a week. This program included care work with the children as well as work with parents/caregivers on their interaction with their children. Both physical and emotional safety of the children was carefully monitored. In addition, children could only be enrolled in therapeutic child care when they had an active child protective services worker appointed from the county to work with the family. The aim of the work of the Center was a successful, positive and safe reunification.

The maltreatment measurers used in this study were coded from the detailed reports of maltreatment in the children’s case files using the Maltreatment Classification System (MCS) (Barnett et al. 1993). This system uses and codes all information available through the records of the Department of Social Services to capture what Cicchetti and Rizley (1981) have described as the ‘heterogeneity of child maltreatment’. Hence the MCS codes all incidents of maltreatment documented by the DSS according to their quantity and characteristics.

The MCS distinguishes four subtypes of maltreatment: sexual abuse, physical abuse, neglect (physical and emotional separately), and emotional maltreatment. Furthermore, it captures the severity of each type of maltreatment from mild neglect or abuse (1) to extremely serious maltreatment (5); the perpetrators if known, the child’s developmental stage at the time of the incident (e.g., infancy, toddlerhood, or preschool), and the frequency of maltreatment incidents (from a single episode (1), to continuous maltreatment, defined as three or more reported incidents (3)). In all maltreated children, the primary caregiver was the perpetrator. Children without any reports of maltreatment were coded 0 on these dimensions, and eligible for the group of non-maltreated children. We also recorded maltreatment frequency and severity. These variables are described in Table 2.

**Table 2:** Descriptions and descriptive statistics for each maltreatment variable

Variable names	Variable values and descriptions	Mean (SD) min-max
Maltreatment status	Dummy variable indicating the child has experienced any type of maltreatment with any severity or frequency. Maltreated = 1; Not maltreated = 0	50%
Maltreatment type	Neglect	21.9%
	Neglect and physical maltreatment (m.)	9.4%
	Neglect and sexual m.	3.1%
	Neglect and emotional m.	18.8%
	Emotional and physical m.	3.1%
	Physical, sexual and emotional m.	3.1%
	neglect, sexual and emotional m.	25%
	Neglect, physical, sexual, and emotional m.	9.4%
	Witness to domestic violence	3.1%
	Unknown type of maltreatment	3.1%
Frequency	Maltreatment frequency is an ordinal variable, with 0 indicating that there has been no maltreatment, and each higher level reflecting a higher number of incidents of abuse: 0 = no reported incident of maltreatment; 1 = single instance; 2 = two reported incidents, episodic; 3 = three or more incidents, continual.	1.2 (1.3)
		0.0–3.0
Severity	Maltreatment severity ranges from no reported incident of maltreatment (=0), to mild abuse or neglect (=1) to extremely serious maltreatment (=5).	1.8 (1.9)
		0.0–5.0

### Data Analyses

We used the software tools offered in the Child Language Data Exchange System (CHILDES) (MacWhinney 1995) for transcribing and coding; CHILDES is an international project which serves to exchange and systematically analyze child language transcripts. After allowing for a warm-up period of 5 min in which the video was not transcribed, the next 25 min (transcribed minutes: 24.6 min, SD 1.5) of each play session tape were transcribed using CHAT (Codes for Human Analysis of Transcripts) software, the transcription tool of CHILDES. As per reliability conventions for transcription, a single transcript was only considered ready for further analysis when checked and verified by a second transcriber. In cases of disagreement, both transcribers revisited the element. Whenever a disagreement on an utterance persisted, or the meaning could not be ascertained, the utterance was coded as unintelligible. Although this study only examines children’s speech, both child and adult talk were transcribed. To generate all general language development and grammatical measures we used the CLAN (Computerized Language ANalysis) software, which provides various analysis tools to perform complex and specific searches across the CHAT files.

We measured all linguistic variables using the CLAN output. All statistical analyses were conducted using SPSS (Released 2007. SPSS for Windows, Version 16.0. Chicago, SPSS Inc.). Mean ± SD is presented for all data. We used one-way analysis of variance (ANOVA) with Bonferroni corrected post-hoc tests where applicable, to investigate group differences. Furthermore, we used linear regression models to examine the impact of each maltreatment variable on general and grammatical language sophistication, and Pearson correlations. The threshold for statistical significance was set at *p* < 0.05.

We examined the distributions of all child language variables in the dataset, examining extreme observations as well as normal probability plots. In most cases, we found distributions very close to normal, with occasional dispersion at the edges of the distribution curves. However, in the normal probability plots we detected one subject that behaved significantly different. Thus, we applied the Jack-knifing technique (Riu and Bro 2003) to look at the influence of the linguistic measures of each subject on each variable in the data set. We investigated whether removing one subject would significantly influence the data. By doing so we detected the same outlier which was excluded from further analyses.

Due to the limited sample size for different types of maltreatment it was not possible to analyze the effects of different maltreatment types on language sophistication. We therefore synthesized a general maltreatment status variable to describe whether the child experienced any kind of maltreatment. We also created a combined variable for maltreatment frequency and severity (i.e., maltreatment intensity: maltreatment frequency times maltreatment severity), as both measures are highly correlated (*r* = 0.919*, p* < 0.01). Maltreatment intensity ranges from a value of 3–15, with an average of 8.75 (±3.25).

## Results

Using a one-way ANOVA, we investigated the differences between maltreated and non-maltreated children (i.e., maltreatment status) across all linguistic measures. The basic comparison on the general language skills variables—talkativeness, lexical diversity, MLU, and TTR—revealed no differences between the groups. As seen in Table 3, the groups were very similar on all general language skills variables. Similar results were found for the linguistic variables that characterize the child’s grammatical sophistication. As shown in Table 3, we only found significant difference in the use of grammatical negations (i.e., grammatical negation/per utterance). Maltreated children used significantly (*df *= 1, *F *= 4.5, *p* = 0.039) more sentence negations (0.07 (±0.05) negations per utterance) than non-maltreated children (0.04 (±0.04) negations per utterance).

**Table 3:** Results of a one-way ANOVA to assess differences between maltreated and non-maltreated children in all measures of general language development and grammatical sophistication measures

	Maltreated	Non-maltreated	Results of one-way ANOVA with Bonferroni adjustment
	Mean (SD)	Mean (SD)	F-value
General language skills			
Talkativeness	14.60 (8.90)	17.58 (10.94)	1.41
Lexical diversity	4.95 (2.41)	5.56 (2.62)	0.95
Types-token ratio	0.37 (0.09)	0.39 (0.15)	0.29
Mean length of utterance	3.13 (0.79)	3.56 (1.0)	3.56
Grammatical sophistication			
Plurals per Token	0.013 (0.014)	0.013 (0.013)	0.00
Unique plurals per types	0.021 (0.017)	0.025 (0.018)	0.72
Third-Person’s-S per token	0.003 (0.003)	0.004 (0.005)	0.57
Past tense	0.062 (0.060)	0.076 (0.099)	0.50
Auxiliaries per token ratio	0.020 (0.018)	0.019 (0.019)	0.09
Negations			
Negation per utterance	0.07 (0.05)	0.04 (0.05)	4.47*
No per token (square root)	0.04 (0.07)	0.02 (0.05)	1.76

*p < .05

In a series of regression analyses we tested the effects of maltreatment intensity on all general and grammatical language sophistication measures, controlling for gender, child age and month in therapy (Table 4). The results show that maltreatment intensity affected significantly only the use of grammatical negations per utterance (*beta *= 0.004, *t *= 2.954, *p* = 0.006). However, all other language sophistication measures remained unaffected by maltreatment intensity, even when controlling for intake age, month in therapy, and gender. Furthermore, we found that intake age positively affected talkativeness (*beta* = 0.649, *t* = 4.773, *p* < 0.001), lexical diversity (*beta *= 0.155, *t *= 4.776, *p* < 0.001), MLU (*beta *= 0.065, *t *= 5.220, *p* < 0.001), third person’s S (*beta *= 0.0001, *t *= 2.216, *p* = 0.033), auxiliaries per token (*beta *= 0.001, *t *= 2.860, *p* = 0.007), and negation per utterance (*beta *= 0.003, *t *= 3.428, *p* = 0.002). It had a negative effect on TTR (*beta *= −0.004, *t* = −2597, *p* = 0.014). Months in therapy also had a positive effect on talkativeness (*beta *= 0.471, *t* = 3.449, *p* = 0.001), lexical diversity (*beta* = 0.139, *t* = 4.276, *p* < 0.001), MLU (*beta* = 0.035, *t *= 2.812, *p* = 0.008), third person’s S (*beta* = 0.0001, *t *= , *p* = 0.), past tense (*beta* = 0.002, *t* = 2.270, *p* = 0.029), and negation per utterance (*beta* = 0.002, *t* = 2.806, *p* = 0.008). Gender never affected the outcome. See Table 4 for details.

**Table 4:** Results of fitted regression models for the effects of maltreatment intensity on all general and grammatical language sophistication measures

	Talkativeness	Lexical diversity	TTR	MLU	Plurals/token	Unique plurals/types	Third P’s S/tokens	Past tense	Auxil./tokens	Negations/ utterance	No/token
B-values											
Constant	−12.584	−1.606	0.531***	0.627	0.009	0.011	−0.003	−0.008	−0.013	−0.077*	0.006
Intake age	0.649***	0.155***	−0.004*	0.065***	0.000	0.000	0.000*	0.002	0.001**	0.003**	0.001
Month in therapy	0.471**	0.139***	−0.002	0.035**	−0.001	0.000	0.000	0.002*	0.000	0.002**	0.000
Gender (Boy = 1)	−1.230	−0.301	0.008	−0.049	0.007	0.003	−0.003	−0.024	0.002	0.004	−0.011
Maltreatment intensity	0.007	−0.023	−0.001	−0.026	−0.000	0.000	−0.001	0.001	0.000	0.004**	0.002
Model fit											
R²	0.47	0.51	0.19	0.54	0.09	0.05	0.16	0.13	0.28	0.39	0.13
F	7.76***	9.20***	2.07	10.34***	0.90	0.46	1.72	1.33	3.39*	5.47**	1.28

*p < 0.05; **p < 0.01; ***p < 0.001

To further investigate the difference in use of negations per utterance we generated a series of three regression models predicting use of negations (Table 5). We began with an unconditional model, and added demographic controls (i.e., intake age, months in therapy, gender). The first model significantly (*df *= 3, *F *= 3.609, *p* = 0.022) explains 23.1% (*r* ^*2*^ *= 0.231*) of the variance in the use of negations per utterance. We found that the use of grammatical negations significantly increases with intake age (*beta* = 0.002, *t *= 3.532, *p* = 0.016) and months in therapy (*beta *= 0.002, *t *= 3.532, *p* = 0.016). We then added controls for language skills (i.e., MLU, TTR, and auxiliaries/tokens) to the second model. Our results show that this second model significantly (*df *= 6, *F *= 2.373, *p* = 0.05) explains 30.1% (*r* ^*2*^ = 0.301) of the variance in the use of negations per utterance. Finally, we added our maltreatment measure. We used the combined maltreatment intensity measure to avoid collinearity, but still account for the correlation of increased maltreatment severity with increased severity. This third model significantly (*df *= 7, *F *= 4.529, *p* = 0.001) explains 49.8% (*r* ^*2*^ = 0.498) of the use of grammatical negations. This model show that maltreatment intensity is the driving factor for use of grammatical negations. The use of grammatical negations significantly increases with maltreatment intensity (*beta* = 0.005, *t *= 3.532, *p* = 0.016), none of the control factors significantly affected the use of grammatical negations per utterance.

**Table 5:** Results of a series of fitted regression models for the effects of maltreatment on negation, controlling for child age, gender, and for MLU, TTR, and use of auxiliaries

	Model 1	Model 2	Model 3
Constant	−0.027	−0.015	−0.118
Child characteristics			
Intake age	0.002*	0.001	0.001
Months in therapy	0.002*	0.001	0.001
Gender (Boy)	0.004	0.004	
Language skills			
MLU		0.013	0.025
TTR		−0.020	0.058
Auxiliaries/tokens		0.456	0.455
Maltreatment			
Intensity			0.005**
Model fit			
R²	0.23	0.30	0.50
F	3.61*	2.37*	4.53**

*p < .05; **p < .01

Although we accounted for age in a regression model, we are aware that our sample has a wide age range. We therefore split our age group at 3.5 years of age (n( ≤ 42 months) = 15; n( > 42 months) = 17) and compared the performances of the two groups on all linguistic measures. In both groups, we only found significant differences on the use of grammatical negations. The younger group ( ≤ 42months) used significantly (*df *= 1, *F* = 4.33, *p* = 0.049) more sentence negations (0.05 (±0.03) negations per utterance) than non-maltreated children (0.02 (±0.02) negations per utterance). The older group ( > 42 months) used significantly (*df *= 1, *F *= 4.17, *p* = 0.049) more sentence negations (0.09 (±0.05) negations per utterance) than non-maltreated children (0.06 (±0.05) negations per utterance).

## Discussion

The purpose of this study was to test whether maltreatment results in a deficit or a difference in the child’s spontaneous language development. The results of the study provide evidence that young maltreated children do not necessarily show a deficit or delay in their language skills when compared to non-maltreated children from the same social background, as seen in the generally equal scores across a set of different language variables. However, the study results imply that maltreated children differ substantially from non-maltreated children in their increased use of grammatical negations during spontaneous speech.

The lack of a general deficit in the language use and grammatical sophistication of maltreated children in the current study was somewhat unexpected, but similar results had been reported before (Alessandri 1991; Flisher et al. 1997; MacFayden and Kitson 1996). Though there were no differences in language skills that were statistically significant, the trend toward significance in the MLU indicates that maltreated children may produce shorter utterances in their spontaneous speech.

One possible explanation for the lack of a deficit in the maltreated children’s language skills is that all children were living in a low socioeconomic environment. Children with low socioeconomic status have been found to have lower language and literary scores at preschool age compared to child with high socioeconomic background (Connell and Prinz 2002; Dieterich et al. 2006). Another explanation for our findings is that both groups of children in our study were enrolled in a therapeutic childcare environment and were receiving services that were intended to support the child in its social development. Though not aimed at remediating language, these services are designed to have a positive effect on a range of social skills as well as cognitive skills, such as language development, for both the maltreated and non-maltreated children participating in this study. Furthermore, the children’s spontaneous language use was scored in an interaction with a caring and safe adult, rather than with a maltreating parent which might lead to a social buffering effect (Cohen and Wills 1985; van Harmelen et al. 2016). In this supportive and less stressful environment the child might feel less threatened, and therefore, more confident when conversing with the peers or the adults.

The results showed that maltreated and non-maltreated children differ substantially in their use of negation. This finding was maintained when splitting the sample into smaller age groups, with a cutoff at 3.5 years of age. The use of sentence negations in spontaneous speech reflects high sophistication in young children’s grammatical skill (Déprez and Pierce 1993; Thornton and Rombough 2015). If the maltreated children in our sample had a general deficit in language, they would also use fewer negations than their non-maltreated peers as use of negations requires a set of sophisticated precursor language skills. Instead, we saw a greater use of negations among the children who had been maltreated in earlier childhood.

One possible reason for the extensive use of grammatical negations may be the development of an overall negativity bias, developed while adapting to the negative experiences in their young lives. Even children younger than 3.5 years of age used double the amount of sentence negations. The negativity biases have also been shown in emotional processing. Günther et al. (2015) found that childhood maltreatment is strongly associated with enhanced attention towards sad emotional stimuli, suggesting a mood-congruent bias which allows enhanced processing of negative emotions. The development of such complex abilities in respect to emotion processing and language skills may possibly enable maltreated children to have a greater self-protective response to and understanding of negative emotions and interpersonal relationships, and furthermore, provide them with a linguistic tool to express more negative self-reflections and perceptions. Relatedly, Ayoub et al. (2006) have elaborated the biasing idea by analyzing children’s (2.5–5 years of age) abilities to represent increasingly sophisticated social concepts. They used a task in which the children were asked to re-tell a series of positively and negatively themed stories. Results showed that although maltreated children had lower levels of complexity than non-maltreated children for the positive stories, they had the same or greater complexity in their stories compared to non-maltreated children when retelling negative stories. Interestingly, the two groups reached similar levels of overall complexity as maltreated children often transformed positive stories into negative versions (Ayoub et al. 2006). This behavior suggests that maltreated children develop a negative bias, which might be shown through negative behaviors, emotions, and cognitive processes.

Moreover, we suggest that the increased use of negations in the structure of language is an early reflection of the overall impact of maltreatment in creating a negative bias in social-cognitive behavior which may show up later in depressive symptoms, internalizing and externalizing behaviors, and an overall negative world view (Ayoub et al. 2006; Gibb and Abela 2008; Tyler et al. 2008, van Harmelen et al. 2010). If a person’s spontaneous language reflects their conceptualization of the world (Carey 2001; Cibelli et al. 2016) and the self (Chen et al. 2014), a maltreated child may express his or her negative world-view not only by negative content but also by using more sentence negations. This would show the interplay between language and thought already during language acquisition (Steinberg et al. 2013). Vice versa, a positive shaping of the language structure could positively impact the world view and the self, which might be relevant for novel therapeutic approaches. Even in early childhood, children’s language reveals the ways that they have adapted to their early experiences. To the attentive adult—caregiver, teacher, therapist, or other interventionist—this can provide insights into the way the child sees the world, and themselves. Thus, language may also provide a means of addressing children’s social-cognitive needs. Vygotsky (1986) proposed that children’s thought processes are socialized via speech that takes place inter-personally, between children and adults or advanced peers, then intra-personally, from the child to themselves as self-talk, then as internal speech. Thus, by modeling a more positive use of language, parents, teachers, or therapists may help children may learn to see the world from a different perspective.

In a recent study, Reece et al. (2017) analyzed written language used in Twitter messages of healthy and depressed individuals. Using predictive computational modelling, they found that the dominate predictor for depression and post-traumatic stress disorder (PTSD) was the use of negative words such as “don’t”, “no” and “never”, as well as some other content words such as “murder” or “death”, used in those messages. This shows that the negative world and negative view of self is deeply imbedded in language and strongly correlated with depressive, post-traumatic and anxiety symptoms. Especially, children who experienced early maltreatment are at elevated risk for developing depression, PTSD and committing suicide in adolescence (for review see Jaffee 2017; Toth et al. 1992). As the use of negative words, or grammatical negations, is already highly increased in early childhood, this indicates the need for early interventions that take speech structure and content into consideration.

As language input shapes a child’s language use (Lany and Saffran 2010), one may also argue that the increased use of grammatical negations as well as one-word negations is instigated by the possibly negatively biased, more neutral and less positive language of the child’s caregiver (Bousha and Twentyman 1984; Dolz et al. 1997; Wilson et al. 2004). However, to date none of the studies presenting that parents use more aversive and neutral commands towards their child also report on the use of grammatical negations or one-word negations (Dolz et al. 1997; Wilson et al. 2004; Teicher et al. 2006). With that in mind, we argue that though the caregiver’s input might have influenced the child’s negative bias, the input likely only aggravates the child’s overall negative world view, leading to an increased use of negations.

Furthermore, our results emphasis the positive effect of supportive and therapeutic interaction, as indicated by a significant performance in increase in relation to a longer time spend in therapy. This finding provides further support for the Buffering Hypothesis (Cohen and Wills 1985), as a social and therapeutic support lead to a positive mediation of negative effects of stress (Evans et al. 2013).

### Limitations

One limitation of the current study is the sample size. Though small samples are common for such a vulnerable and hard to enroll population, a larger sample may reveal some of the subtle differences in the effects of different types of maltreatment that the current sample could not do. However, all cases of maltreatment have been officially confirmed.

Being aware that not all cases of maltreatment are officially reported (Stoltenborgh et al. 2015), we need to take in consideration that this might be the reason for the homogeneity in the language development. In order to assess the effect of the therapeutic service it would also be of interest to assess the language abilities of a group of children from the same preschool who did not receive the services.

The current sample had a large age range. Also, the current sample was very young, therefore, we cannot rule out the possibility that language deficits occur over time.

We did not specifically elicit the maximum language skills from each child through a series of language tasks. The naturalistic setting of this study, observing the children in context of a therapist and child, is perhaps more capable of revealing the negativity bias in everyday linguistic interactions. However, analyzing the language of the parents and/or the therapists could shed light on how language input shapes the language of maltreated children.
